# Artificial intelligence can extract important features for diagnosing axillary lymph node metastasis in early breast cancer using contrast-enhanced ultrasonography

**DOI:** 10.1038/s41598-025-90099-9

**Published:** 2025-02-15

**Authors:** Tomohiro Oshino, Ken Enda, Hirokazu Shimizu, Megumi Sato, Mutsumi Nishida, Fumi Kato, Yoshitaka Oda, Mitsuchika Hosoda, Kohsuke Kudo, Norimasa Iwasaki, Shinya Tanaka, Masato Takahashi

**Affiliations:** 1 Department of Breast Surgery, Faculty of Medicine and Graduate School of Medicine, Hokkaido University, Sapporo, Hokkaido Japan; 2 Department of Cancer Pathology, Faculty of Medicine and Graduate School of Medicine, Hokkaido University, Sapporo, Hokkaido Japan; 3 Department of Orthopaedic Surgery, Faculty of Medicine and Graduate School of Medicine, Hokkaido University, Sapporo, Hokkaido Japan; 4 Diagnostic Center for Sonography, Hokkaido University Hospital, Sapporo, Japan; 5 Department of Diagnostic and Interventional Radiology, Hokkaido University Hospital, Sapporo, Japan; 6 Department of Radiology, Jichi Medical University Saitama Medical Center, Saitama, Saitama Japan; 7 Department of Breast Surgery, Hokkaido University Hospital, Kita 14 Nishi 5, Kita-ku, Sapporo, Hokkaido Japan; 8 Department of Diagnostic Imaging, Graduate School, Faculty of Medicine, Hokkaido University, Sapporo, Japan; 9 Medical AI Research and Developmental Center, Hokkaido University Hospital, Sapporo, Japan; 10 Institute for Chemical Reaction Design and Discovery (WPI-ICReDD), Hokkaido University, Sapporo, Hokkaido Japan

**Keywords:** Breast cancer, Cancer imaging

## Abstract

Contrast-enhanced ultrasound (CEUS) plays a pivotal role in the diagnosis of primary breast cancer and in axillary lymph node (ALN) metastasis. However, the imaging features that are clinically crucial for lymph node metastasis have not been fully elucidated. Hence, we developed a bimodal model to predict ALN metastasis in patients with early breast cancer by integrating CEUS images with the annotated imaging features. The model adopted a light-gradient boosting machine to produce feature importance, enabling the extraction of clinically crucial imaging features. In this retrospective study, the diagnostic performance of the model was investigated using 788 CEUS images of ALNs obtained from 788 patients who underwent breast surgery between 2013 and 2021, with the ground truth defined by the pathological diagnosis. The results indicated that the test cohort had an area under the receiver operating characteristic curve (AUC) value of 0.93 (95% confidence interval: 0.88, 0.98). The model had an accuracy of 0.93, which was higher than the radiologist’s diagnosis (accuracy of 0.85). The most important imaging features were heterogeneous enhancement, diffuse cortical thickening, and eccentric cortical thickening. Our model has an excellent diagnostic performance, and the extracted imaging features could be crucial for confirming ALN metastasis in clinical settings.

**Supplementary Information:**

The online version contains supplementary material available at 10.1038/s41598-025-90099-9.

## Introduction

Breast cancer is the most common malignancy in women and has the second-highest mortality rate^1,2^. Axillary lymph node (ALN) metastasis in early breast cancer affects prognosis^3,4^. Both axillary dissection and sentinel lymph node biopsy, performed to evaluate and treat ALN in stage 0–3 early breast cancer, are invasive and unacceptable complications persist^5^. Therefore, in recent years, ALN surgery for breast cancer has been de-escalating, and case selection for ALN surgical reduction often requires imaging evaluation of the ALN to confirm the absence of suspected metastases^6,7^. Therefore, an accurate and non-invasive method for predicting the ALN status without axillary surgery is warranted.

Conventional ultrasonography (US) is the method of choice for assessing ALN metastasis in patients with known breast cancer^8,9^, with a sensitivity ranging from 69 to 85% and a specificity of 55–72%^10–12^ for non-palpable lymph nodes. This limited diagnostic performance is due to the limited imaging of deep ALNs and the inability to show typical morphological changes^13^, which has prevented the use of conventional US in a wide variety of applications^14^. Compared with conventional US, contrast-enhanced ultrasonography (CEUS) can substantially improves the visualisation of vascularity and parenchymal microcirculation within the tumour by the injection of a microbubble contrast agent^14^. In fact, CEUS improves the diagnostic performance for predicting ALN metastasis compared with conventional US, with a sensitivity of 61–100% and specificity of 82–100%^10,15–18^. Furthermore, CEUS contrast agents such as Sonazoid^®^^19^ have a low risk of allergies and are safe with a very low incidence of side effects^20^. Currently, CEUS plays a pivotal role in the diagnosis of lymph node metastasis^21^.

Therefore, CEUS is routinely performed in patients with early stage breast cancer at our facility. However, many diagnostic indicators^22^ as well as standard imaging and image interpretation methods have not yet been established, resulting in low penetration rates. To make CEUS universal and effective, crucial imaging features must be extracted from CEUS images.

Machine learning (ML) models are vital for providing optimal solutions and revealing complex interrelationships between virables^23^. Previous studies have stated that tree-based models outperform deep learning (DL) models on tabular data^24^, because imaging features are regarded as tabular data. A light-gradient boosting machine (LightGBM) with feature selection, a representative tree-based model, has been widely applied for evaluating tabular datasets and extracting feature importance for each parameter^25–28^. Attempts have also been made to predict ALN metastasis in breast cancer based on conventional US images of primary breast cancer tumour^29^. However, to date, no reports have analysed CEUS using these ML models have been published.

Based on this hypothesis, important features can be extracted in CEUS using ML models. Therefore, we developed a bimodal model to predict ALN metastasis in patients with early breast cancer by integrating CEUS images with annotated imaging features. This study aimed to evaluate the performance of this model and to extract crucial features for predicting ALN metastasis.

## Materials and methods

### Patients and study design

**Fig. 1:**
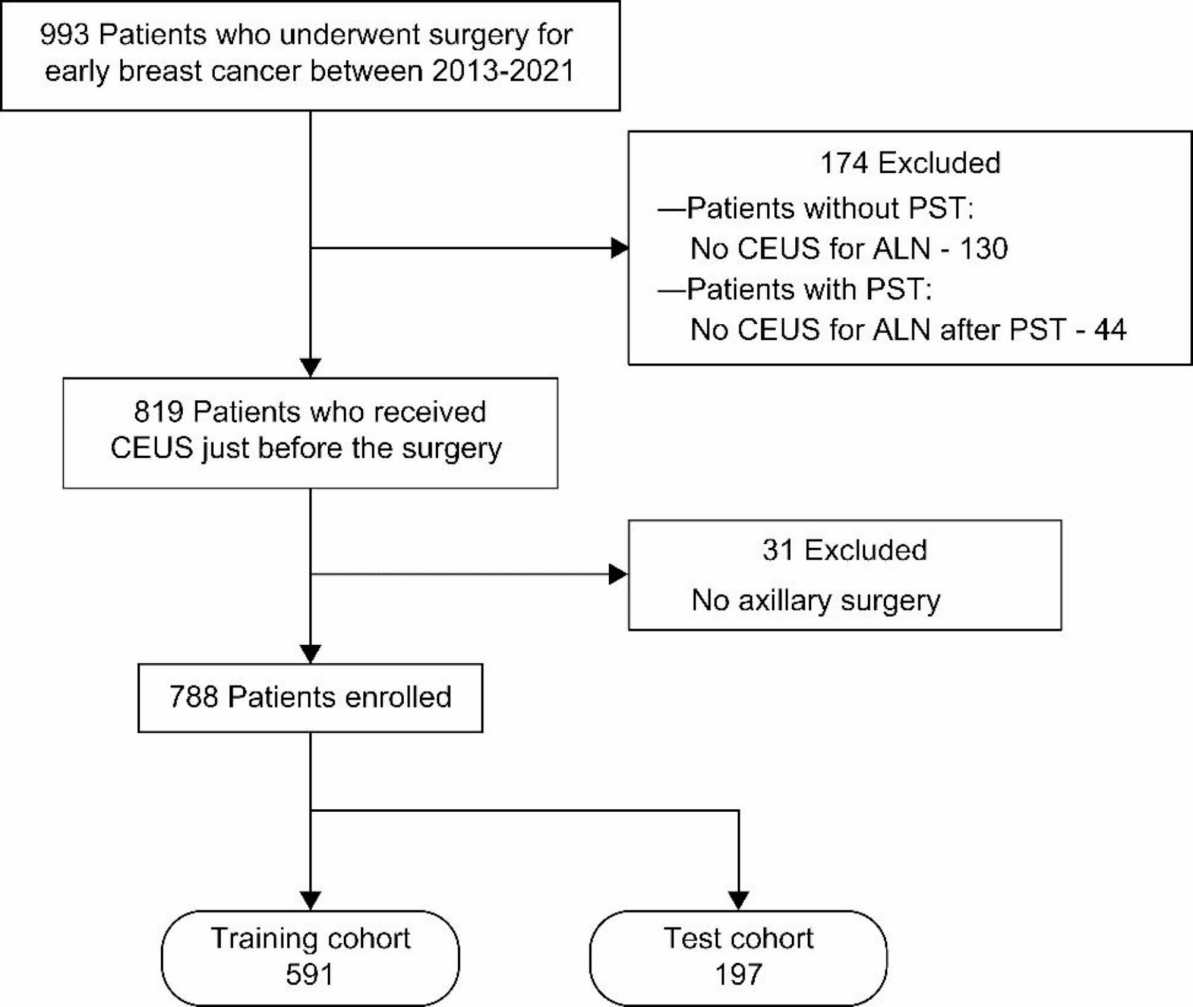
Patient flowchart. *ALN* axillary lymph node, *CEUS* contrast-enhanced ultrasonography, *PST* preoperative systemic therapy.

In this single-centre retrospective study, we reviewed the records of 993 patients who underwent surgery for stage 0–3 early breast cancer at our hospital (Fig. 1). If the patient had received preoperative systemic therapy, CEUS, including chemotherapy and endocrine therapy, was required. After excluding patients who had no ALN evaluated using CEUS and/or no axillary surgery, we enrolled 788 patients in this study. The ground truth was defined as the pathological diagnosis of ALN metastasis.

Among the 788 patients (mean age 59.7 [range, 25–90] years), 182 (23%), 331 (42%), 247 (31%), and 28 (4%) patients were categorised as clinical stage (cStage) 0, 1, 2, and 3, respectively. A total of 190 patients (24%) had ALN metastases. The enrolled patients were stratified by the pathological diagnosis of ALN metastasis and randomly divided into training and independent test sets in a ratio of 3:1. Clinicopathological characteristics including breast imaging reporting and data system (BI-RADs) category, subtypes, and the Ki67 labelling index are shown in Table 1,

**Table 1:** Patient characteristics.

Characteristics	Training cohort (*n* = 591)	Test cohort (*n* = 197)	Whole cohort (*n* = 788)	*p*-value
Age (years, mean ± SD)	59.6 ± 0.531	59.9 ± 0.87	59.7 ± 0.45	0.74
Preoperative systemic therapy				0.11
Yes	144 (24%)	54 (27%)	198 (25%)	
No	447 (76%)	143 (73%)	590 (75%)	
Conventional US size of primary breast tumour (mm, mean ± SD)	13.4 ± 0.504	15.0 ± 0.944	13.8 ± 0.45	0.17
BI-RADS category				0.33
4A	157 (27%)	57 (29%)	214 (27%)	
4B	92 (16%)	31 (16%)	123 (16%)	
4C	99 (17%)	41 (21%)	140 (18%)	
5	154 (26%)	39 (20%)	193 (24%)	
Oestrogen receptor				0.32
Positive	471 (80%)	166 (84%)	637 (81%)	
Negative	120 (20%)	31 (16%)	151 (19%)	
Progesterone receptor				0.55
Positive	416 (70%)	141 (72%)	557 (71%)	
Negative	174 (29%)	56 (28%)	230 (29%)	
HER2				0.005
Positive	103 (17%)	23 (12%)	126 (16%)	
Negative or equivocal	486 (82%)	173 (88%)	659 (84%)	
Ki67*	27.6 ± 0.981	27.8 ± 1.67	27.6 ± 0.845	0.81
Tumour type				0.19
Ductal carcinoma in situ	65 (11%)	20 (10%)	85 (11%)	
Invasive ductal carcinoma	436 (74%)	140 (71%)	576 (73%)	
Invasive lobular carcinoma	31 (5%)	16 (8%)	47 (6%)	
Other types	38 (6%)	16 (8%)	54 (7%)	

Data are presented as the number of patients with percentages in parentheses unless noted otherwise.

*BI-RADS* breast imaging reporting and data system, *HER2* human epidermal growth factor receptor 2, *SD* standard deviation.

### US protocols and radiologist assessments

Preoperative conventional US and CEUS of breast cancer and ALN were performed by two sonographers specialising in breast US and CEUS with five years of experience. CEUS was performed for ALNs most suspicious for metastasis, as detected by conventional US. CEUS was performed with Sonazoid^®^ (GE HealthCare Pharma, Tokyo, Japan) 0.015 mL/kg/body by intravenous injection, and the primary breast tumour and ALN were evaluated in the maximal contrast accumulation cross-section. The ALN images with the highest contrast accumulation in the CEUS series were selected for this study. Contrast accumulation reached its peak in two distinct phases: the arterial phase, approximately 20 s after contrast injection, and the late phase, approximately 180–200 s after injection.

The conventional US and CEUS images were discussed by two radiologists with 24 and 29 years of experience, who were blinded to the pathological data, and a consensus assessment was completed in each case.

### Framework of the proposed model

**Fig. 2:**
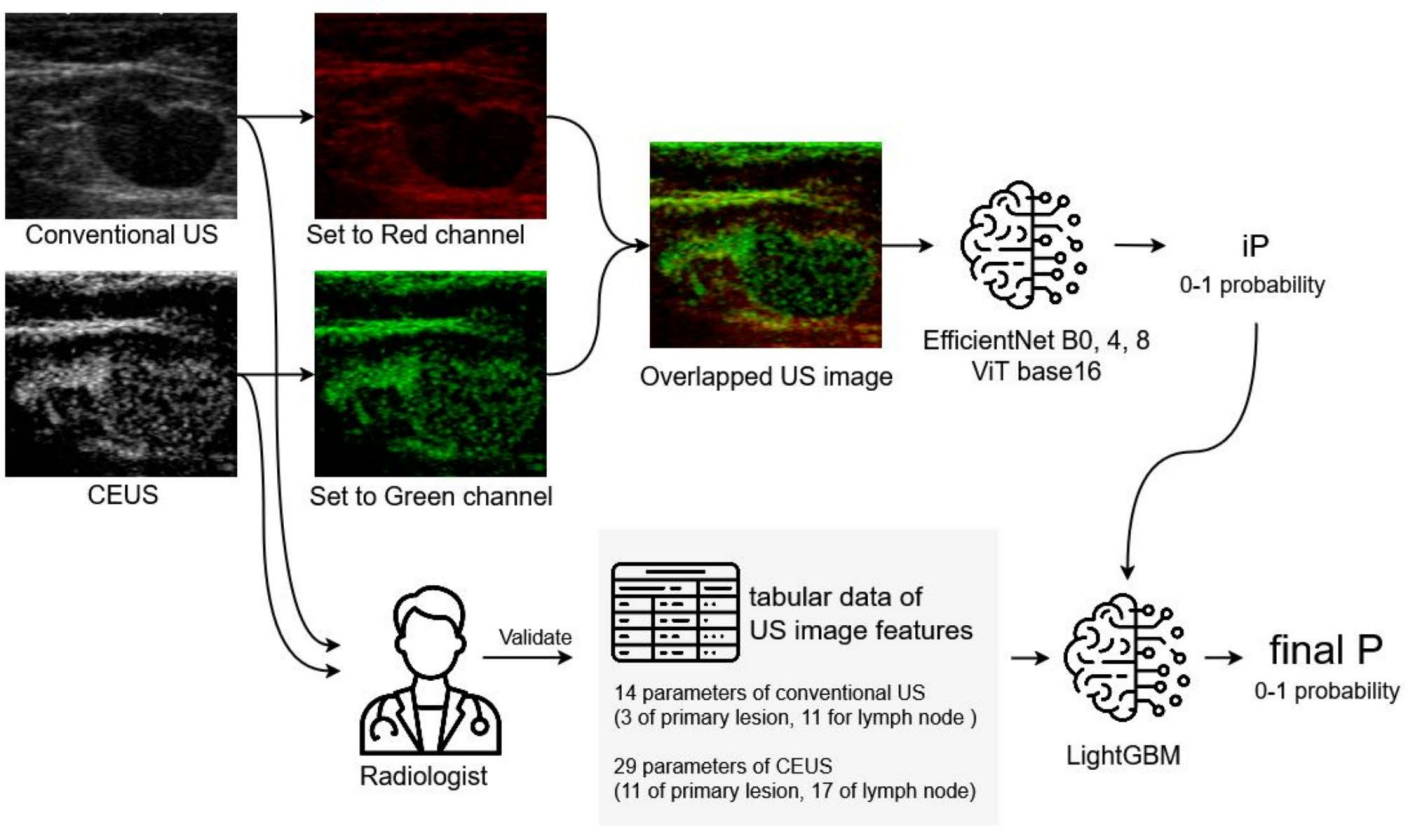
Overview of our bimodal model consisted of DL part and LightGBM part. The DL contained image preprocessing steps, where the RGB channels were overlaid by putting the high-echo areas of the conventional US image in R and the contrast areas of the CEUS image in G. Then, the image was input into DL models to derive the image-based predictive covariate (iP: 0–1) for ALN metastasis. In the LightGBM part input data were iP and imaging features which radiologists validated in conventional US and CEUS images of ALN and primary lesions. The output was defined as final predictive covariate (final P). The performance of models were compared in the two settings. Setting A had input data from conventional US images and the imaging features. Setting B had input data from conventional US images combined with CEUS images and the imaging features. *DL deep learning*,* LightGBM* light-gradient boosting machine, *CEUS* contrast-enhanced ultrasonography, *US* ultrasonography, *ALN* axillary lymph node.

Our bimodal model consisted of a DL component for US images and a ML component for tabular data (Fig. 2). The input data in the DL part were conventional US and CEUS images. Following the image preprocessing steps, DL models were used to derive image-based predictive covariates (iP: 0–1) for ALN metastasis. In the ML component, the input data for LightGBM were as follows: iP and imaging features validated by radiologists in conventional US and CEUS images of ALN and primary lesions. The output was defined as the final predictive covariate (final P). The performance of the models was also evaluated using only conventional US images and the corresponding imaging features.

### The DL part

#### Image preprocessing

The examination generated DICOM data containing paired conventional US and CEUS images for each patient. These DICOM images were converted to standard rasterised images, and regions of interest from both conventional US and CEUS images were cropped and resized to 512 × 512 pixels (384 × 384 pixels for VisionTransformer models) and normalised based on the mean and standard colours.

Both images were converted to greyscale using ITU-R BT.709 coefficients (Y = 0.2989R + 0.5870G + 0.1140B). For the final RGB composite image, the grayscale conventional US image was assigned to the R channel, the grayscale CEUS image to the G channel, and the B channel was set to zero. These composite images were subsequently used for DL analysis with a low computational cost^30^ to derive an image-based predictive covariate (iP: 0–1).

#### DL analysis on CEUS and conventional US images

We implemented several architectures: EfficientNet^31^ B0, B4, B8, and ViT^32^ Base/16, all of which were pre-trained on ImageNet and imported from the PyTorch image model library^33^. During training, image augmentation was applied as follows: random resizing/cropping, horizontal flipping, noise addition, and colour arrangement^34^, to mitigate overfitting and sample imbalances.

For hyperparameter optimisation, the training set was further split into training and validation sets in a 4:1 ratio. After the optimal hyperparameters were determined using the internal validation set, the final model was trained using the entire training set. Each model was trained using binary cross-entropy as the loss function, with sigmoid activation. Training was conducted using the Adam optimizer^35^ with a learning rate of 1e-3, batch size of two, and 30 epochs.

### The ML part

#### Imaging features in CEUS and conventional US

All imaging features were evaluated by two breast surgery specialists with 9 and 34 years of experience and subsequently validated by radiologists. Interobserver agreement was tested by evaluating 80 randomly selected patients. Specific numerical values are listed in Supplementary Table S1. The positivity rates for each US feature in the training and test cohorts are listed in Supplementary Table S2.

For primary breast tumours, conventional US assessment included the BI-RADS category and long and short diameters of the invasive lesions. The CEUS evaluation of primary tumours comprised the long and short diameters of invasive lesions and nine specific findings^36^: (1) enhanced time compared with surrounding breast tissue, (2) enhanced intensity compared with surrounding tissue, (3) enhanced direction, (4) internal homogeneity of the lesion, (5) margin of the lesion after enhancement, (6) shape of the lesion, (7) ring-like enhancement, (8) scope of the lesion (maximal diameter of the lesion in the CEUS image with that in conventional US image), and (9) perfusion defect.

For ALNs, conventional US assessment consisted of the long and short diameters and the following nine features (t1–9)^29^: (1) ratio of long axis diameter to short axis diameter < 2, (2) diffuse cortical thickening > 3 mm, (3) focal cortical bulge > 3 mm, (4) eccentric cortical thickening > 3 mm, (5) complete or partial effacement of the fatty hilum, (6) rounded hypoechoic node, (7) complete or partial replacement of the node with an ill-defined or irregular mass, (8) microcalcifications in the node, and (9) nonhilar cortical blood flow on colour Doppler images.

CEUS evaluation of ALN included the nine features (t1–9) mentioned above^29^, and the following six features (B1–B6)^37^:1) centripetal enhancement, 2) heterogeneous enhancement pattern, 3) perfusion defect, 4) ring enhancement, 5) enlarged enhancement range on CEUS compared to conventional US, and 6) microcalcification.

The features were converted into a tabular format by quantifying them as binary (0 or 1), categorical (including 2–7 in some cases), or scalar measurements such as lesion diameters.

#### LightGBM and feature selection

Tabular data analysis incorporated two types of input features: DL-derived iP and imaging features. The LightGBM model parameters were optimised as follows: number of leaves = 31, maximum depth = 3, minimum data in leaf = 20, learning rate = 0.1, and early stopping round = 50. For the training cohort, five-fold cross-validation was implemented during training to minimise overfitting, resulting in five distinct models. During the training, feature selection was conducted as the feature importance of each tabular data point was quantified.

#### Evaluation metrics

Model performance was evaluated using the test set. The model output (final P) underwent a logistic transformation to constrain the predicted probabilities between 0 and 1. The performance was evaluated by comparing the final P with the ground truth labels at different thresholds using the following metrics:

$${\text{Sensitivity }}={\text{ }}\left( {{\text{true positive}}} \right){\text{ }}/{\text{ }}({\text{true positive}}\,+\,{\text{false negative}})$$

$${\text{Specificity }}={\text{ }}\left( {{\text{true negative}}} \right){\text{ }}/{\text{ }}({\text{true negative}}\,+\,{\text{false positive}})$$

$${\text{Positive predictive value }}={\text{ }}\left( {{\text{true positive}}} \right){\text{ }}/{\text{ }}({\text{true positive}}\,+\,{\text{false positive}})$$

$${\text{Negative predictive value }}={\text{ }}\left( {{\text{true negative}}} \right){\text{ }}/{\text{ }}({\text{true negative}}\,+\,{\text{false negative}})$$

$${\text{Accuracy }}={\text{ }}({\text{true positive}}\,+\,{\text{true negative}}){\text{ }}/{\text{ }}\left( {{\text{all samples}}} \right)$$

### Statistical analysis

Detailed clinical and pathological differences between the training and test cohorts were compared using the t-test or Mann-Whitney U test. The kappa test was used to compare the interobserver agreement. The DeLong test was used to compare the two receiver operating characteristic curves and compare ML and clinical diagnoses. All statistical analyses were two-sided, and a *p*-value < 0.05 indicated statistical significance.

### Software and implementation

DL analyses were performed using Python 3.12.8 and PyTorch 2.3.0. Tabular data analyses were performed using LightGBM 4.3.0. Statistical analyses were performed using Bell Curve for Excel 4.04 and R 4.3.1.

## Results

### The diagnostic performance in the DL part

EfficientNet B0, B4, B8, and VisionTransformer (ViT) Base/16 were used to determine the best DL model. The area under the receiver operating characteristic curve (AUROC) of EfficientNet B8 was 0.69, which was the highest score among the four models (Table 2). Therefore, the output of EfficientNet B8 was defined as the image-based predictive covariate (iP).

**Table 2:** Diagnostic performance of DL model input CEUS image for predicting ALN metastasis on the test cohort.

	AUC	Accuracy	Sensitivity	Specificity
EfficientNet B0	0.667	0.679	0.553	0.727
EfficientNet B4	0.685	0.637	0.642	0.638
EfficientNet B8	0.687	0.716	0.553	0.776
ViT Base/16	0.673	0.768	0.404	0.895

*AUC* area under the receiver operating characteristics curve. *ViT* Vision Transformer.

### The diagnostic performance of the proposed bimodal model

The bimodal model had AUROC of 0.93 (0.88–0.98), with a sensitivity of 0.88, a specificity of 0.96, and an accuracy of 0.93 at the Youden index point. The kappa values for evaluating tabular formatted dataset findings on the whole cohort were 0.96 (0.94–0.97) for inter-observer agreement (*p* < 0.001, kappa test).

**Fig. 3:**
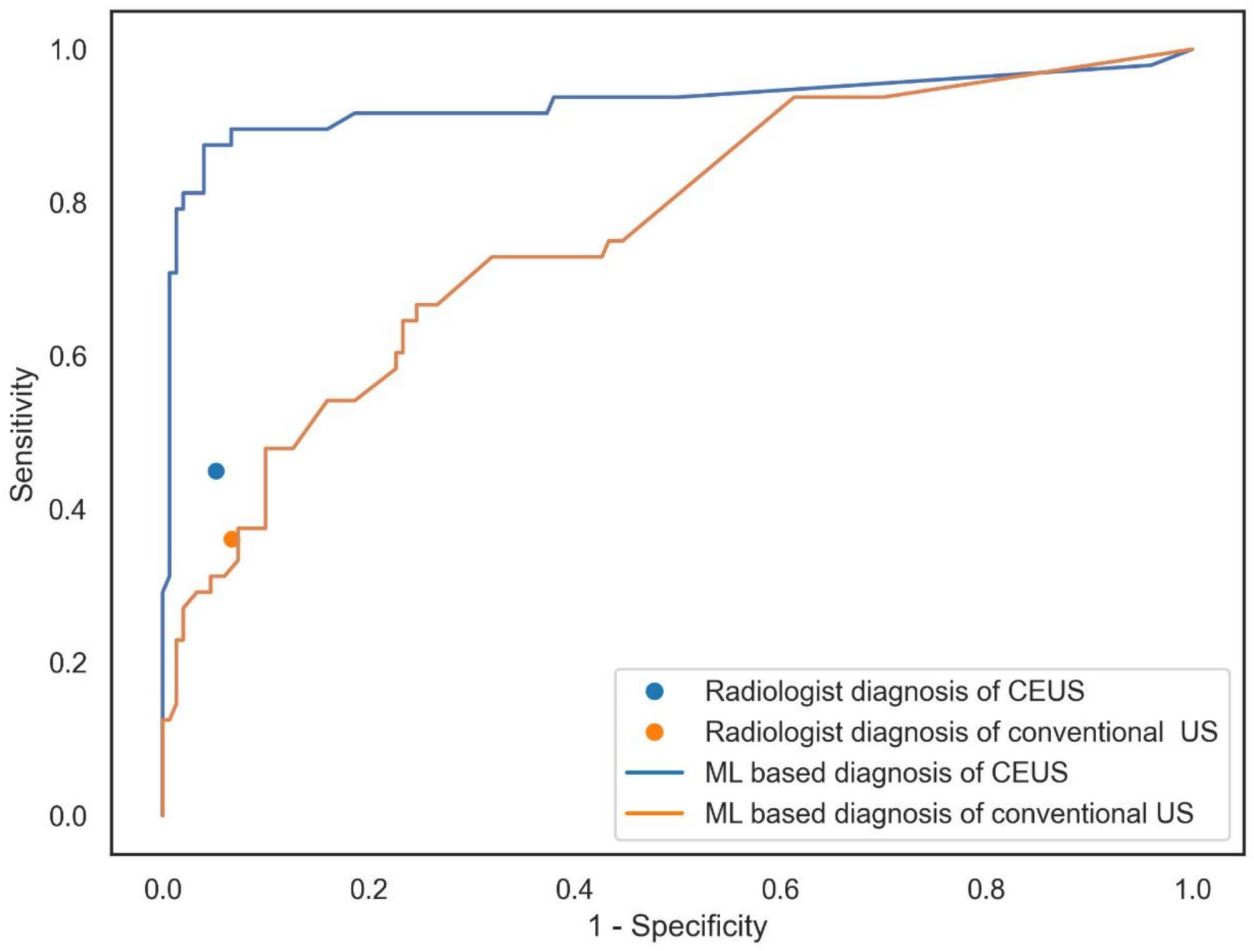
Diagnostic performance of our proposed model and the radiologist’s diagnosis for predicting ALN metastasis in the test cohort. *US* ultrasonography, *CEUS* contrast-enhanced ultrasonography.

The model with use of conventional US images alone and their correspondent imaging features alone had AUROC of 0.76 (0.68–0.85) (Fig. 3), with a sensitivity of 0.67, a specificity of 0.75, and an accuracy of 0.77 at the Youden index point, demonstrating adding CEUS images and the image features improve the diagnostic performance in our model (*p* < 0.001, DeLong test). The diagnostic performance of the bimodal model based on CEUS, iP, and conventional US findings is presented in Table 3.

**Table 3:** Diagnostic performance of LightGBM models for predicting ALN metastasis in the test cohort.

	AUC	Accuracy	Sensitivity	Specificity
CEUS + iP + cUS	0.93 (0.88–0.98)	0.93	0.88	0.95
CEUS	0.93 (0.87–0.98)	0.92	0.88	0.95
iP	0.64 (0.55–0.73)	0.63	0.60	0.64
cUS	0.76 (0.66–0.85)	0.72	0.67	0.73

*AUC* area under the receiver operating characteristics curve, *CEUS* contrast-enhanced ultrasonography, *cUS* conventional ultrasonography, *iP* the image-based predictive covariate (0–1).

**Fig. 4:**
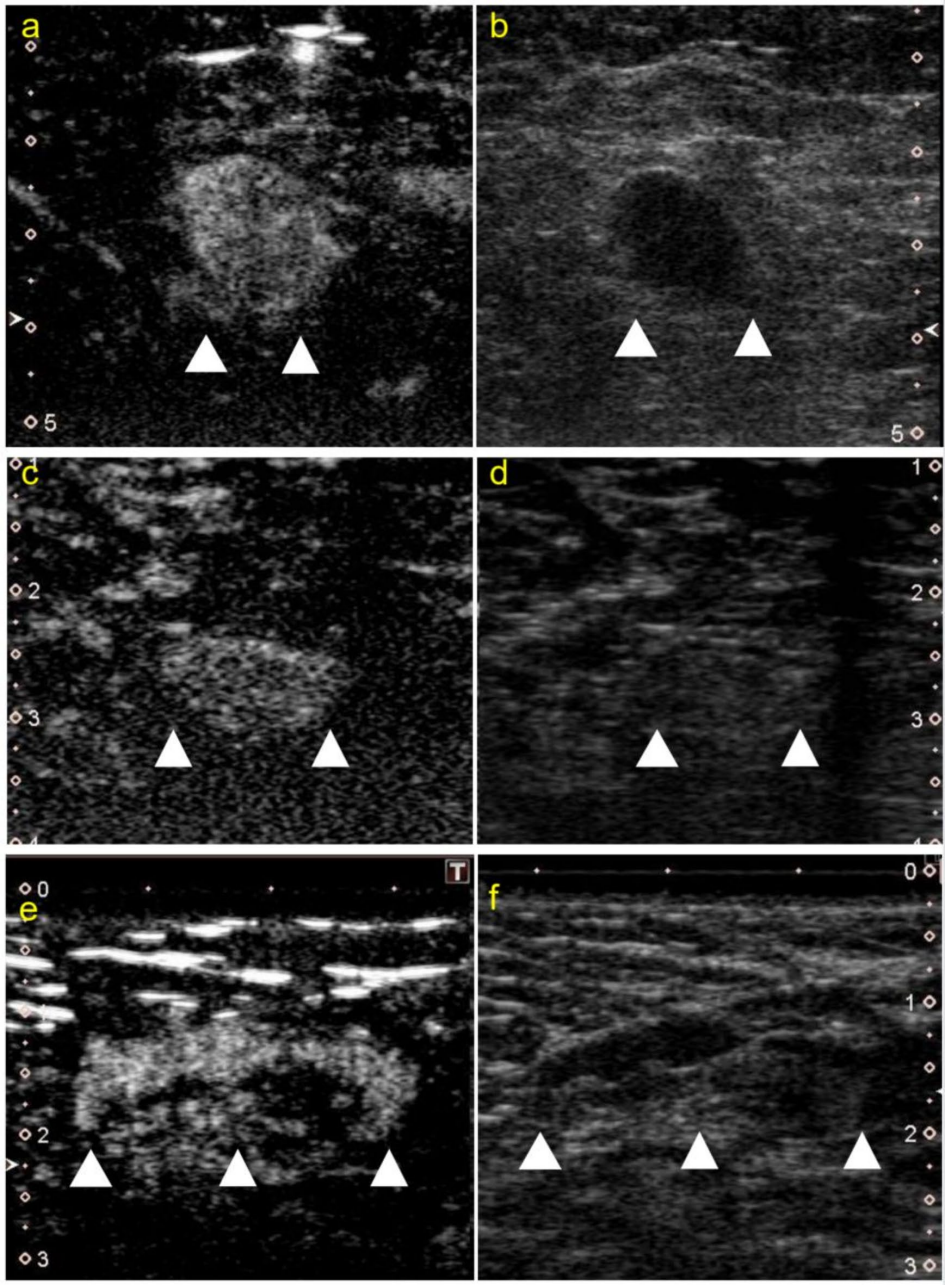
Representative images of the CEUS and conventional US images (**a**,**b**) CEUS (**a**) and conventional US (**b**) images with ground truth positive; radiologist’s diagnosis positive; ML model positive. (**c**,**d**) CEUS (**c**) and conventional US (**d**) images with ground truth positive; radiologist’s diagnosis negative; ML model positive. (**e**,**f**) CEUS (**e**) and conventional US (**f**) images with ground truth negative; Radiologist’s diagnosis positive; ML model negative. *US* ultrasonography, *CEUS* contrast-enhanced ultrasonography, *ML* machine learning.

The radiologist’s diagnosis had a sensitivity of 0.36, specificity of 0.93, and accuracy of 0.82. The CEUS examination had a sensitivity of 0.45, and a specificity of 0.95, and accuracy of 0.85 for the test cohort (Fig. 3). The radiologist’s accuracy was lower than that of the proposed model in both settings. Representative US images are shown in Fig. 4.

### Feature importance values

The feature importance values extracted using the LightGBM are listed in Table 4. In order of importance were B2 (heterogeneous enhancement pattern) at 3.24 × 10^2^, t2 (diffuse cortical thickening > 3 mm) at 3.16 × 10^2^, t4 (eccentric cortical thickening > 3 mm) at 2.58 × 10^2^, invasive diameter of primary breast tumour (CEUS) at 1.54 × 10^2^, and B5 (enlarged enhancement range on CEUS than in conventional US) at 1.46 × 10^2^. iP had the sixth highest feature importance at 5.14 × 10^1^.

**Table 4:** Top six highly weighted features in terms of importance extracted with the LightGBM using CEUS images and tabular formatted data of each US finding.

	Features	Importance
1	B2: Heterogeneous enhancement pattern (CEUS)	3.24 × 10^2^
2	t2: Diffuse cortical thickening > 3 mm (CEUS)	3.16 × 10^2^
3	t4: Eccentric cortical thickening > 3 mm (CEUS)	2.58 × 10^2^
4	Invasive diameter of primary breast tumour (CEUS)	1.54 × 10^2^
5	B5: Enlarged enhancement range on CEUS compared to cUS	1.46 × 10^2^
6	iP (CEUS imaging)	5.14 × 10^1^

*CEUS* contrast-enhanced ultrasonography, *iP* image-based predictive covariate (0–1), *cUS* conventional ultrasonography.

### Diagnostic performance using the imaging features with high feature importance

Diagnostic performance was validated using imaging features with the highest importance: B2, t2, and t4. When one or more positive features were observed among the three features, it had a sensitivity of 0.68, a specificity of 0.95, and an accuracy of 0.90 for the test cohort.

## Discussion

Our bimodal model using CEUS had excellent diagnostic performance for predicting ALN metastasis and extracted features that are important for diagnosis. The clinical significance of the extracted features with high importance was consistent with that of previous reports^38,39^.

Precise evaluation of ALN is important for determining a treatment plan for early stage breast cancer; however, the burden of examination should be considered. US imaging has distinct advantages over magnetic resonance imaging or computed tomography, including a lower cost and higher frequency of use^40^. In practice, magnetic resonance imaging findings are followed up with US and a US-guided biopsy is performed^41^. Although the diagnostic performance of conventional US is limited^13,14^, the present study suggests the possibility of solving this problem. Our study supports the use of CEUS images to develop bimodal models that can potentially be powerful tools for predicting lymph node metastasis with clinical utility in decision-making guidance^40^.

The dynamic images can be used to further refine the proposed model. We evaluated the prediction of ALN metastasis without the use of dynamic imaging or imaging features. Dynamic enhancement patterns, such as peak intensity^14,15^, time to peak^15^, and contrast duration^15^, have been reported to improve CEUS diagnoses. We did not include features from dynamic images because only a small number of cases underwent dynamic analysis of the ALN to prioritise the evaluation of primary breast cancer. However, dynamic input images may further improve the diagnostic performance. Recently, a report used still and movie clips of thyroid US as input data, with movie clips being superior to still images^42^. For further studies, collecting dynamic images as input data may improve the diagnostic performance by adding dynamic images and their findings.

Although ML models for CEUS images have the potential to be innovative, there are issues, especially with regard to US images, such as image quality assessment and standardised imaging protocols. The value of US examination in breast cancer is limited by operator dependency^43^, and standardisation is more difficult to achieve than for other imaging modalities^43,44^. To address these issues, a clinical trial recently began with a clear and standardised protocol and criteria for US imaging of ALN^45^. Thus, ML models of US images for ALN could be developed to confirm whether the images are suitable for use in diagnosis in the future.

In the clinical perspective, the present study showed the radiologist’s diagnosis with a sensitivity of 0.45 and specificity of 0.95 in CEUS. In conventional US examination, the diagnosis had a sensitivity of 0.36 and specificity of 0.93. As US examinations were performed in patients with early breast cancer, the assessments focused on confirming the diagnosis of ALN metastasis. These clinical requirements may reflect the results with high specificity and low sensitivity.

A clinical report also showed that the conventional US findings of ALN metastasis differed between invasive lobular carcinoma and invasive ductal carcinoma^46^. This indicates the substantial possibility that CEUS images of ALN metastasis could be subdivided based on the subtypes. While our study did not perform partitioning, further studies should be conducted with larger datasets.

Our study had some limitations. First, this retrospective single-centre study did not include an external validation cohort. Therefore, there may have been a selection or recall bias. Further studies should be performed prospectively in multiple centres to validate the model performance. Second, there was potential model overfitting, although five-fold cross-validation was implemented during training in the ML part, and some augmentations were performed in the DL part. Therefore, it is necessary to analyse larger datasets in the future. Third, the fact that the features were determined by the physicians’ judgement indicates that this was not an end-to-end study, which is a concern owing to bias.

In conclusion, our ensemble model evaluated the conventional US and CEUS features of primary breast tumours and ALNs and extracted important CEUS features for the diagnosis of ALN metastasis.

## Electronic supplementary material

Below is the link to the electronic supplementary material.

Supplementary Material 1

## Data Availability

The data supporting the findings of this study are available from the corresponding author upon request.

## Code availability

The implementation code is publicly available for both DL analysis (https://github.com/endaaman/br-image) and LightGBM on tabular data (https://github.com/endaaman/br-clinical).
