# Supplementary material for: Artificial intelligence can extract important features for diagnosing axillary lymph node metastasis in early breast cancer using contrast-enhanced ultrasonography
Source: Sci Rep. 2025 Feb 15;15:5648. doi: 10.1038/s41598-025-90099-9 (PMC11829987; doi:10.1038/s41598-025-90099-9)
Supplement: Supplementary file 1 — Supplementary Material 1 [file 41598_2025_90099_MOESM1_ESM.docx]

| **Long and short diameter of primary breast tumour and axillary lymph node** | | | | | | |  |
| --- | --- | --- | --- | --- | --- | --- | --- |
| Qualitative variables are in (mm) | | | | | | |  |
| **BI-RADS category by conventional US** | | |  |  |  |  |  |
| BI-RADS category 1 | | 1 |  |  |  |  |  |
| BI-RADS category 2 | | 2 |  |  |  |  |  |
| BI-RADS category 3 | | 3 |  |  |  |  |  |
| BI-RADS category 4A | | 4 |  |  |  |  |  |
| BI-RADS category 4B | | 5 |  |  |  |  |  |
| BI-RADS category 4C | | 6 |  |  |  |  |  |
| BI-RADS category 5 | | 7 |  |  |  |  |  |
|  | **Primary breast tumour by CEUS** | | | | | | |
| (1) | Enhanced time compared with surrounding breast tissue | | | | | | |
|  | (synchronous or later = 0, earlier = 1) | | | | | | |
| (2) | Enhanced intensity compared with surrounding tissue | | | | | | |
|  | (not enhanced = 0, enhanced = 1) | | | | | | |
| (3) | Enhanced direction | | | | | | |
|  | (diffuse = 0, centripetal = 1, centrifugal = 2) | | | | | | |
| (4) | Internal homogeneity of the lesion | | | | | | |
|  | (homogeneous = 0, heterogeneous = 1) | | | | | | |
| (5) | Margin of the lesion after enhancement | | | | | | |
|  | (clear = 0, not clear = 1) | | | | | | |
| (6) | Shape of the lesion | | | | | | |
|  | (regular = 0, irregular = 1) | | | | | | |
| (7) | Ring-like enhancement | | | | | | |
|  | (negative = 0, positive = 1) | | | | | | |
| (8) | Scope of the lesion (maximal diameter of the lesion in CEUS image vs conventional US) | | | | | | |
|  | (negative = 0, positive = 1) | | | | | | |
| (9) | Perfusion defect positive | | | | | | |
|  | (negative = 0, positive = 1) | | | | | | |
| **t 1-9** | **Axillary lymph node findings by conventional US and CEUS (negative = 0, positive = 1)** | | | | | | |
| t1 | Ratio of long axis diameter to short axis diameter <2 | | | | | | |
| t2 | Diffuse cortical thickening >3 mm | | | | | | |
| t3 | Focal cortical bulge >3 mm | | | | | | |
| t4 | Eccentric cortical thickening >3 mm | | | | | | |
| t5 | Complete or partial effacement of the fatty hilum | | | | | | |
| t6 | Rounded hypoechoic node | | | | | | |
| t7 | Complete or partial replacement of the node with an ill-defined or irregular mass | | | | | | |
| t8 | Microcalcifications in the node | | | | | | |
| t9 | Nonhilar cortical blood flow on colour Doppler images | | | | | | |
| **B1-B6** | **Axillary lymph node findings by only CEUS (negative = 0, positive = 1)** | | | | | | |
| B1 | Centripetal enhancement | | | | | | |
| B2 | Heterogenous enhancement pattern | | | | | | |
| B3 | Perfusion defect | | | | | | |
| B4 | Ring enhancement | | | | | | |
| B5 | Enlarged enhancement range on CEUS compared to conventional US | | | | | | |
| B6 | Microcalcification | | | | | | |
|  | | | | | | |  |
| **Target label data (axillary lymph node metastasis):** The ground truth | | | | | | |  |
| **Axillary lymph node metastasis** | | | **Diagnosis** | | |  |  |
| No axillary lymph node metastasis | | | pN0 | | | 0 |  |
| Any axillary lymph node metastasis | | | pN+ (1mi,1a,1b,1c,2a,2b,2c,3a,3b,3c) | | | 1 |  |

**Supplemental Table S1.** Tabular-formatted dataset findings. *BI-RADS* breast imaging reporting and data system; *CEUS* contrast-enhanced ultrasonography.

| **Features** | **Training cohort**  **(n = 591)** | **Test cohort**  **(n = 197)** | ***p*-value** |
| --- | --- | --- | --- |
| **Conventional US** |  |  |  |
| t1 positive | 82 (14%) | 24 (12%) | 0.56 |
| t2 positive | 22 (4%) | 9 (5%) | 0.59 |
| t3 positive | 84 (14%) | 30 (15%) | 0.71 |
| t4 positive | 29 (5%) | 7 (4%) | 0.44 |
| t5 positive | 22 (4%) | 7 (4%) | 0.92 |
| t6 positive | 41 (7%) | 13 (7%) | 0.88 |
| t7 positive | 15 (3%) | 4 (2%) | 0.69 |
| t8 positive | 15 (3%) | 3 (2%) | 0.41 |
| t9 positive | 31 (5%) | 8 (4%) | 0.51 |
| **CEUS** |  |  |  |
| t1 positive | 79 (13%) | 28 (14%) | 0.76 |
| t2 positive | 50 (9%) | 20 (10%) | 0.47 |
| t3 positive | 68 (12%) | 24 (12%) | 0.80 |
| t4 positive | 55 (9%) | 25 (13%) | 0.17 |
| t5 positive | 16 (3%) | 8 (4%) | 0.34 |
| t6 positive | 37 (6%) | 13 (7%) | 0.87 |
| t7 positive | 15 (3%) | 4 (2%) | 0.69 |
| t8 positive | 16 (3%) | 3 (2%) | 0.35 |
| t9 positive | 56 (10%) | 20 (10%) | 0.78 |
|  |  |  |  |
| B1 positive | 71 (12%) | 27 (14%) | 0.53 |
| B2 positive | 86 (15%) | 33 (17%) | 0.46 |
| B3 positive | 54 (9%) | 21 (11%) | 0.53 |
| B4 positive | 3 (1%) | 1 (1%) | 1.00 |
| B5 positive | 71 (12%) | 28 (14%) | 0.42 |
| B6 positive | 9 (2%) | 2 (1%) | 0.60 |

**Supplemental Table S2.** Positivity rate for each US feature. *CEUS* contrast-enhanced ultrasonography.
